# *trans*-Diaqua­bis­(l-phenyl­alaninato-κ^2^ *N*,*O*)nickel(II)

**DOI:** 10.1107/S160053681201080X

**Published:** 2012-03-17

**Authors:** Massomeh Ghorbanloo, Nahid Shahbakhsh, Duane Choquesillo-Lazarte

**Affiliations:** a Department of Chemistry, University of Zanjan, 45371-38791 Zanjan, Iran; b Laboratorio de Estudios Cristalográficos, IACT, CSIC-Universidad de Granada, Av. de las Palmeras 4, 18100 Armilla, Granada, Spain

## Abstract

In the title compound, [Ni(C_9_H_10_NO_2_)_2_(H_2_O)_2_], the coordination geometry around the Ni^II^ ion can be described as distorted octa­hedral, with two N atoms and two O atoms from phenyl­alaninate ligands in the basal plane and two aqua O atoms at the axial sites. The crystal packing is stabilized by inter­molecular O—H⋯O and N—H⋯O hydrogen bonds.

## Related literature

For background to amino acid complexes, see: Thanavelan *et al.* (2011 ▶). For related structures, see: Rombach *et al.* (2002 ▶); Marandi & Shahbakhsh (2007 ▶). For similar hydrogen-bonded networks, see: Cao *et al.* (2011 ▶). For details of π–π stacking inter­actions, see: Janiak (2000) ▶.
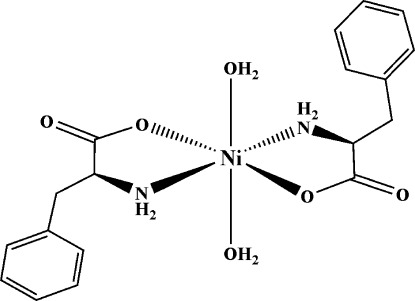


## Experimental

### 

#### Crystal data

[Ni(C_9_H_10_NO_2_)_2_(H_2_O)_2_]
*M* *_r_* = 423.10
Monoclinic, 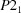

*a* = 4.8272 (5) Å
*b* = 32.617 (4) Å
*c* = 6.0585 (7) Å
β = 105.995 (1)°
*V* = 916.97 (18) Å^3^
*Z* = 2
Mo *K*α radiation
μ = 1.10 mm^−1^
*T* = 100 K
0.46 × 0.15 × 0.15 mm

#### Data collection

Bruker SMART APEX diffractometer
Absorption correction: multi-scan (*SADABS*; Bruker, 2008 ▶) *T*_min_ = 0.633, *T*_max_ = 0.853
8826 measured reflections
3214 independent reflections
3157 reflections with *I* > 2σ(*I*)
*R*_int_ = 0.020

#### Refinement

*R*[*F* ^2^ > 2σ(*F* ^2^)] = 0.023
*wR*(*F* ^2^) = 0.058
*S* = 1.06
3214 reflections
256 parameters
5 restraints
H atoms treated by a mixture of independent and constrained refinement
Δρ_max_ = 0.43 e Å^−3^
Δρ_min_ = −0.22 e Å^−3^
Absolute structure: Flack (1983 ▶), 1567 Friedel pairs
Flack parameter: −0.003 (10)

### 

Data collection: *APEX2* (Bruker, 2010 ▶); cell refinement: *SAINT* (Bruker, 2010 ▶); data reduction: *SAINT*; program(s) used to solve structure: *SHELXS97* (Sheldrick, 2008 ▶); program(s) used to refine structure: *SHELXL97* (Sheldrick, 2008 ▶); molecular graphics: *ORTEP-3* (Farrugia, 1997 ▶); software used to prepare material for publication: *publCIF* (Westrip, 2010 ▶).

## Supplementary Material

Crystal structure: contains datablock(s) I, global. DOI: 10.1107/S160053681201080X/br2191sup1.cif

Structure factors: contains datablock(s) I. DOI: 10.1107/S160053681201080X/br2191Isup2.hkl

Additional supplementary materials:  crystallographic information; 3D view; checkCIF report

## Figures and Tables

**Table 1:** Hydrogen-bond geometry (Å, °)

*D*—H⋯*A*	*D*—H	H⋯*A*	*D*⋯*A*	*D*—H⋯*A*
O13—H13*A*⋯O1^i^	0.84 (2)	1.95 (2)	2.747 (2)	159 (2)
O13—H13*B*⋯O23^ii^	0.86 (2)	1.88 (2)	2.658 (2)	150 (3)
O33—H33*A*⋯O3^iii^	0.83 (2)	1.88 (2)	2.691 (2)	163 (3)
O33—H33*B*⋯O21^iv^	0.83 (2)	1.97 (2)	2.748 (2)	156 (2)
N5—H5*B*⋯O1^i^	0.92	2.49	3.359 (2)	157
N5—H5*A*⋯O3^iii^	0.92	2.39	3.193 (3)	147
N25—H25*A*⋯O13^iv^	0.92	2.57	3.148 (2)	122
N25—H25*A*⋯O21^iv^	0.92	2.47	3.310 (2)	153
N25—H25*B*⋯O23^ii^	0.92	2.36	3.181 (3)	149

Symmetry codes: (i) 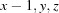; (ii) 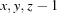; (iii) 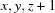; (iv) 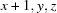.

**Fig. 1.**
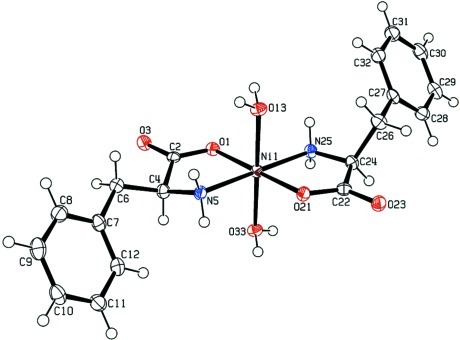
The molecular structure of the title compound, with atom labels and anisotropic displacement ellipsoids (drawn at 50% probability level) for non-H atoms.

**Fig. 2.**
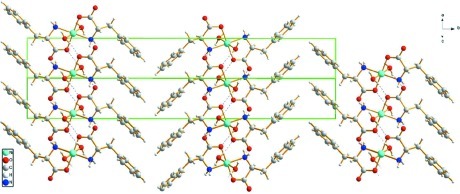
The packing diagram of the title compound which O–H···O hydrogen bonds shown as blue dashed lines.

## supplementary crystallographic information

### Comment

Amino acids are of special importance among the other chemical substances since they form the basic constituents of living organisms. It is imperative to know the properties of amino acids in order to understand and explain their behavior and the synthesis of peptides, proteins and enzymes in living organisms. Also they are widely applied in food, cosmetic, pharmaceutical and chemical industry. It is known that the reactions of peptides, proteins and enzymes with metal ions are of biochemical importance but they are yet to be thoroughly understood (Thanavelan *et al.*, 2011). The explanation of these phenomena in the biological systems can be possible only by the determination of structure of amino acids.

Because of the importance the characterization of amino acid derivatives, here, we report the synthesis and crystal structure of Trans-diaqua-bis[(L-phenylalanine)-*κ^2^*N,O]nickel(II). In the title compound, [Ni(OH_2_)_2_(C_18_H_20_N_2_O_4_)_2_], the coordination geometry around the nickel(II) can be described as a distored octahedral which is shown in Fig. 1. In the title compound, the amino acid ligands form equatorial plane and axial positions are occupied by the oxygen atoms from aqua ligands. The oxygen atoms of the amino acid ligands are located *trans* to each other. Moreover, the nitrogen atom of the amino acid ligand (N5) is located *trans* to the nitrogen atom of the other amino acid (N25). In the title compound the amino acid ligands form two five–membered chelate rings.

The carboxylate groups of the amino acids in the title compound are involved in anti–anti bidentate bridging coordination. The amino acid is N,O-chelated, forming a five-membered ring. Unlike our complex, most of aminoacid complexes with this kind of O,N chelation form coordination polymers held together by bridging carboxylate ligands (Rombach *et al.*, 2002).

This configuration is stabilized by four intermolecular hydrogen bonds of the types O—H···O=C—O and O—H···O—C=O and five hydrogen bonds of the type N—H···O=C—O and N—H···O—C=O (Fig. 2). The carboxylate groups are the acceptors of all hydrogen bonds. Really, this structure as composed of molecules linked by hydrogen bonded into layers leading to 1D network.

### Experimental

All reagents were commercially available and used as received. For preparing the title compound a methanol (10 ml) solution of L-phenylalanine (2 mmol) and NaOH (2mmol) were added to a methanol solution (10 ml) of Ni(NO_3_)_2_.6H_2_O (1 mmol), and the mixture was refluxed for 6 h.

The X-ray quality blue crystals of the title compound were obtained by slow solvent evaporation during 5 days. Yield: 68%, mp > 400 °C. IR (cm^-1^): 3357 (broad, H_2_O), 1594 (vs, *ν*as(COO)), 1497 (s, *ν*s(COO)), 1404 (s, *δ*-NH_2_), 450 (w), 546 (w), 575 (w).

### Refinement

H atoms were located in difference Fourier maps and included in the refinement as constrained idealized atoms riding on the parent atom, with C-H = 0.95 Å (aromatic groups), 1.00 Å (CH–N groups), 0.99 Å (CH_2_–Ph groups) or 0.92 Å (–NH_2_ groups) and with *U*_iso_(H) = 1.2*U*_eq_(C,N). The H atoms of the aqua ligands were refined as semi-free with a distance restraint, and with *U*_iso_(H) = 1.2*U*_eq_(O).

### Crystal data

**Table:**

[Ni(C_9_H_10_NO_2_)_2_(H_2_O)_2_]	*F*(000) = 444
*M**_r_* = 423.10	*D*_x_ = 1.532 Mg m^−^^3^
Monoclinic, *P*2_1_	Mo *K*α radiation, λ = 0.71073 Å
Hall symbol: P 2yb	Cell parameters from 7357 reflections
*a* = 4.8272 (5) Å	θ = 2.5–27.8°
*b* = 32.617 (4) Å	µ = 1.10 mm^−^^1^
*c* = 6.0585 (7) Å	*T* = 100 K
β = 105.995 (1)°	Block, pale blue
*V* = 916.97 (18) Å^3^	0.46 × 0.15 × 0.15 mm
*Z* = 2	

### Data collection

**Table:**

Bruker SMART APEX diffractometer	3214 independent reflections
Radiation source: fine-focus sealed tube	3157 reflections with *I* > 2σ(*I*)
Graphite monochromator	*R*_int_ = 0.020
φ and ω scans	θ_max_ = 25.0°, θ_min_ = 1.3°
Absorption correction: multi-scan (*SADABS*; Bruker, 2008)	*h* = −5→5
*T*_min_ = 0.633, *T*_max_ = 0.853	*k* = −38→38
8826 measured reflections	*l* = −7→7

### Refinement

**Table:**

Refinement on *F*^2^	Secondary atom site location: difference Fourier map
Least-squares matrix: full	Hydrogen site location: inferred from neighbouring sites
*R*[*F*^2^ > 2σ(*F*^2^)] = 0.023	H atoms treated by a mixture of independent and constrained refinement
*wR*(*F*^2^) = 0.058	*w* = 1/[σ^2^(*F*_o_^2^) + (0.0344*P*)^2^ + 0.0026*P*] where *P* = (*F*_o_^2^ + 2*F*_c_^2^)/3
*S* = 1.06	(Δ/σ)_max_ < 0.001
3214 reflections	Δρ_max_ = 0.43 e Å^−^^3^
256 parameters	Δρ_min_ = −0.22 e Å^−^^3^
5 restraints	Absolute structure: Flack (1983), 1567 Friedel pairs
Primary atom site location: structure-invariant direct methods	Flack parameter: −0.003 (10)

### Special details

**Table:**

Geometry. All esds (except the esd in the dihedral angle between two l.s. planes) are estimated using the full covariance matrix. The cell esds are taken into account individually in the estimation of esds in distances, angles and torsion angles; correlations between esds in cell parameters are only used when they are defined by crystal symmetry. An approximate (isotropic) treatment of cell esds is used for estimating esds involving l.s. planes.
Refinement. Refinement of *F*^2^ against ALL reflections. The weighted *R*-factor *wR* and goodness of fit *S* are based on *F*^2^, conventional *R*-factors *R* are based on *F*, with *F* set to zero for negative *F*^2^. The threshold expression of *F*^2^ > σ(*F*^2^) is used only for calculating *R*-factors(gt) *etc*. and is not relevant to the choice of reflections for refinement. *R*-factors based on *F*^2^ are statistically about twice as large as those based on *F*, and *R*- factors based on ALL data will be even larger.

### Fractional atomic coordinates and isotropic or equivalent isotropic displacement parameters (Å^2^)

**Table:**

	*x*	*y*	*z*	*U*_iso_*/*U*_eq_	
Ni1	0.28611 (5)	0.648490 (11)	0.40794 (4)	0.01552 (8)	
O1	0.4353 (3)	0.62803 (5)	0.1433 (3)	0.0176 (3)	
C2	0.3680 (5)	0.59137 (6)	0.0787 (4)	0.0154 (5)	
O3	0.4153 (4)	0.57545 (5)	−0.0940 (3)	0.0208 (4)	
C4	0.2425 (5)	0.56442 (7)	0.2377 (4)	0.0187 (5)	
H4	0.4108	0.5512	0.3495	0.022*	
N5	0.1012 (4)	0.59095 (5)	0.3755 (3)	0.0166 (4)	
H5A	0.1239	0.5796	0.5184	0.020*	
H5B	−0.0929	0.5929	0.3038	0.020*	
C6	0.0588 (5)	0.52992 (7)	0.1089 (4)	0.0213 (5)	
H6A	0.1709	0.5153	0.0189	0.026*	
H6B	−0.1116	0.5421	−0.0019	0.026*	
C7	−0.0470 (5)	0.49844 (7)	0.2534 (4)	0.0212 (5)	
C8	−0.2788 (5)	0.47360 (8)	0.1455 (4)	0.0245 (5)	
H8	−0.3693	0.4773	−0.0136	0.029*	
C9	−0.3811 (6)	0.44351 (9)	0.2643 (5)	0.0285 (6)	
H9	−0.5387	0.4268	0.1864	0.034*	
C10	−0.2533 (6)	0.43785 (8)	0.4970 (5)	0.0262 (6)	
H10	−0.3241	0.4175	0.5794	0.031*	
C11	−0.0218 (6)	0.46217 (7)	0.6083 (4)	0.0249 (5)	
H11	0.0675	0.4584	0.7675	0.030*	
C12	0.0812 (5)	0.49241 (7)	0.4865 (4)	0.0233 (5)	
H12	0.2402	0.5089	0.5640	0.028*	
O13	−0.0828 (4)	0.67531 (5)	0.1807 (3)	0.0190 (3)	
H13A	−0.231 (4)	0.6608 (7)	0.133 (4)	0.023*	
H13B	−0.042 (6)	0.6837 (8)	0.059 (3)	0.023*	
O21	0.1315 (3)	0.66878 (5)	0.6664 (3)	0.0173 (3)	
C22	0.2192 (5)	0.70390 (7)	0.7484 (4)	0.0170 (5)	
O23	0.1545 (4)	0.72004 (5)	0.9143 (3)	0.0215 (4)	
C24	0.4021 (5)	0.72964 (7)	0.6278 (4)	0.0177 (5)	
H24	0.5846	0.7379	0.7437	0.021*	
N25	0.4761 (4)	0.70600 (5)	0.4425 (3)	0.0169 (4)	
H25A	0.6730	0.7032	0.4760	0.020*	
H25B	0.4138	0.7200	0.3060	0.020*	
C26	0.2290 (5)	0.76835 (7)	0.5355 (4)	0.0218 (5)	
H26A	0.0524	0.7601	0.4164	0.026*	
H26B	0.1680	0.7813	0.6623	0.026*	
C27	0.3862 (5)	0.80002 (7)	0.4338 (4)	0.0198 (5)	
C28	0.6211 (5)	0.82146 (7)	0.5734 (4)	0.0220 (5)	
H28	0.6866	0.8153	0.7326	0.026*	
C29	0.7596 (6)	0.85182 (8)	0.4812 (5)	0.0255 (6)	
H29	0.9187	0.8662	0.5773	0.031*	
C30	0.6654 (6)	0.86095 (8)	0.2499 (5)	0.0274 (7)	
H30	0.7605	0.8815	0.1868	0.033*	
C31	0.4317 (6)	0.84014 (8)	0.1094 (4)	0.0278 (6)	
H31	0.3651	0.8467	−0.0492	0.033*	
C32	0.2955 (5)	0.80967 (7)	0.2019 (4)	0.0239 (5)	
H32	0.1377	0.7952	0.1046	0.029*	
O33	0.6519 (4)	0.62138 (5)	0.6364 (3)	0.0187 (3)	
H33A	0.592 (6)	0.6108 (8)	0.740 (4)	0.022*	
H33B	0.792 (4)	0.6368 (6)	0.684 (4)	0.022*	

### Atomic displacement parameters (Å^2^)

**Table:**

	*U*^11^	*U*^22^	*U*^33^	*U*^12^	*U*^13^	*U*^23^
Ni1	0.01495 (13)	0.01567 (13)	0.01611 (13)	−0.00181 (13)	0.00456 (9)	0.00010 (13)
O1	0.0157 (8)	0.0181 (8)	0.0192 (8)	−0.0020 (6)	0.0051 (6)	0.0016 (7)
C2	0.0127 (11)	0.0169 (12)	0.0149 (12)	0.0017 (9)	0.0011 (9)	0.0025 (9)
O3	0.0238 (9)	0.0194 (8)	0.0224 (9)	−0.0007 (7)	0.0120 (7)	0.0004 (7)
C4	0.0191 (12)	0.0182 (12)	0.0197 (12)	−0.0010 (9)	0.0067 (10)	−0.0008 (9)
N5	0.0181 (10)	0.0161 (10)	0.0156 (9)	−0.0020 (8)	0.0044 (8)	−0.0015 (7)
C6	0.0250 (13)	0.0195 (12)	0.0186 (12)	−0.0010 (10)	0.0048 (10)	−0.0007 (9)
C7	0.0235 (13)	0.0177 (12)	0.0253 (13)	0.0015 (10)	0.0118 (10)	−0.0026 (9)
C8	0.0250 (13)	0.0251 (13)	0.0228 (13)	0.0014 (10)	0.0054 (10)	−0.0018 (10)
C9	0.0239 (15)	0.0243 (14)	0.0378 (16)	−0.0079 (11)	0.0092 (12)	−0.0063 (12)
C10	0.0332 (16)	0.0180 (13)	0.0329 (15)	−0.0034 (11)	0.0184 (13)	−0.0009 (11)
C11	0.0310 (14)	0.0197 (12)	0.0251 (13)	0.0021 (11)	0.0098 (11)	0.0016 (10)
C12	0.0234 (13)	0.0182 (12)	0.0279 (13)	−0.0034 (10)	0.0065 (11)	−0.0037 (10)
O13	0.0169 (8)	0.0235 (9)	0.0168 (9)	−0.0037 (7)	0.0051 (7)	0.0035 (7)
O21	0.0198 (8)	0.0170 (8)	0.0162 (8)	−0.0034 (7)	0.0069 (7)	−0.0011 (6)
C22	0.0131 (11)	0.0197 (12)	0.0174 (12)	0.0010 (9)	0.0030 (9)	0.0026 (9)
O23	0.0298 (10)	0.0194 (9)	0.0180 (9)	−0.0024 (7)	0.0111 (7)	0.0001 (7)
C24	0.0178 (12)	0.0155 (11)	0.0207 (12)	−0.0008 (9)	0.0068 (10)	−0.0002 (9)
N25	0.0171 (10)	0.0159 (10)	0.0184 (10)	−0.0017 (8)	0.0064 (8)	−0.0004 (7)
C26	0.0200 (12)	0.0211 (12)	0.0260 (12)	0.0023 (10)	0.0093 (10)	0.0026 (10)
C27	0.0215 (12)	0.0148 (11)	0.0253 (13)	0.0037 (10)	0.0101 (10)	−0.0005 (9)
C28	0.0233 (12)	0.0212 (12)	0.0221 (12)	0.0036 (10)	0.0076 (10)	0.0026 (9)
C29	0.0227 (14)	0.0170 (13)	0.0383 (16)	0.0007 (11)	0.0108 (12)	0.0000 (12)
C30	0.0340 (16)	0.0176 (13)	0.0367 (17)	0.0022 (11)	0.0200 (13)	0.0041 (11)
C31	0.0401 (16)	0.0232 (13)	0.0238 (13)	0.0040 (11)	0.0153 (12)	0.0032 (10)
C32	0.0292 (13)	0.0198 (12)	0.0236 (13)	0.0010 (10)	0.0086 (11)	0.0000 (10)
O33	0.0164 (8)	0.0219 (9)	0.0175 (8)	−0.0040 (7)	0.0042 (7)	0.0027 (6)

### Geometric parameters (Å, º)

**Table:**

Ni1—O21	2.0223 (16)	C12—H12	0.9500
Ni1—O1	2.0421 (16)	O13—H13A	0.839 (17)
Ni1—N5	2.0642 (18)	O13—H13B	0.861 (17)
Ni1—N25	2.0731 (18)	O21—C22	1.274 (3)
Ni1—O33	2.1139 (17)	C22—O23	1.249 (3)
Ni1—O13	2.1171 (17)	C22—C24	1.541 (3)
O1—C2	1.272 (3)	C24—N25	1.484 (3)
C2—O3	1.245 (3)	C24—C26	1.532 (3)
C2—C4	1.546 (3)	C24—H24	1.0000
C4—N5	1.492 (3)	N25—H25A	0.9200
C4—C6	1.510 (3)	N25—H25B	0.9200
C4—H4	1.0000	C26—C27	1.510 (3)
N5—H5A	0.9200	C26—H26A	0.9900
N5—H5B	0.9200	C26—H26B	0.9900
C6—C7	1.526 (3)	C27—C32	1.388 (3)
C6—H6A	0.9900	C27—C28	1.401 (3)
C6—H6B	0.9900	C28—C29	1.395 (4)
C7—C8	1.390 (3)	C28—H28	0.9500
C7—C12	1.391 (3)	C29—C30	1.381 (4)
C8—C9	1.386 (4)	C29—H29	0.9500
C8—H8	0.9500	C30—C31	1.389 (4)
C9—C10	1.386 (4)	C30—H30	0.9500
C9—H9	0.9500	C31—C32	1.392 (3)
C10—C11	1.385 (4)	C31—H31	0.9500
C10—H10	0.9500	C32—H32	0.9500
C11—C12	1.402 (3)	O33—H33A	0.833 (17)
C11—H11	0.9500	O33—H33B	0.829 (17)
			
O21—Ni1—O1	179.03 (7)	C12—C11—H11	119.9
O21—Ni1—N5	97.42 (7)	C7—C12—C11	120.7 (2)
O1—Ni1—N5	82.24 (7)	C7—C12—H12	119.6
O21—Ni1—N25	82.71 (7)	C11—C12—H12	119.6
O1—Ni1—N25	97.64 (7)	Ni1—O13—H13A	118.2 (18)
N5—Ni1—N25	179.38 (8)	Ni1—O13—H13B	109.7 (18)
O21—Ni1—O33	92.85 (6)	H13A—O13—H13B	105 (2)
O1—Ni1—O33	88.04 (7)	C22—O21—Ni1	116.26 (14)
N5—Ni1—O33	86.75 (7)	O23—C22—O21	124.3 (2)
N25—Ni1—O33	92.64 (7)	O23—C22—C24	117.1 (2)
O21—Ni1—O13	86.82 (6)	O21—C22—C24	118.5 (2)
O1—Ni1—O13	92.29 (6)	N25—C24—C26	111.88 (18)
N5—Ni1—O13	92.84 (7)	N25—C24—C22	111.37 (18)
N25—Ni1—O13	87.77 (7)	C26—C24—C22	107.21 (18)
O33—Ni1—O13	179.43 (7)	N25—C24—H24	108.8
C2—O1—Ni1	115.66 (14)	C26—C24—H24	108.8
O3—C2—O1	124.1 (2)	C22—C24—H24	108.8
O3—C2—C4	118.74 (19)	C24—N25—Ni1	110.76 (14)
O1—C2—C4	116.99 (19)	C24—N25—H25A	109.5
N5—C4—C6	115.3 (2)	Ni1—N25—H25A	109.5
N5—C4—C2	109.68 (17)	C24—N25—H25B	109.5
C6—C4—C2	112.15 (19)	Ni1—N25—H25B	109.5
N5—C4—H4	106.4	H25A—N25—H25B	108.1
C6—C4—H4	106.4	C27—C26—C24	115.33 (19)
C2—C4—H4	106.4	C27—C26—H26A	108.4
C4—N5—Ni1	109.17 (14)	C24—C26—H26A	108.4
C4—N5—H5A	109.8	C27—C26—H26B	108.4
Ni1—N5—H5A	109.8	C24—C26—H26B	108.4
C4—N5—H5B	109.8	H26A—C26—H26B	107.5
Ni1—N5—H5B	109.8	C32—C27—C28	118.4 (2)
H5A—N5—H5B	108.3	C32—C27—C26	121.0 (2)
C4—C6—C7	116.5 (2)	C28—C27—C26	120.6 (2)
C4—C6—H6A	108.2	C29—C28—C27	120.7 (2)
C7—C6—H6A	108.2	C29—C28—H28	119.7
C4—C6—H6B	108.2	C27—C28—H28	119.7
C7—C6—H6B	108.2	C30—C29—C28	119.9 (3)
H6A—C6—H6B	107.3	C30—C29—H29	120.0
C8—C7—C12	118.1 (2)	C28—C29—H29	120.0
C8—C7—C6	118.4 (2)	C29—C30—C31	120.1 (2)
C12—C7—C6	123.5 (2)	C29—C30—H30	120.0
C9—C8—C7	121.6 (2)	C31—C30—H30	120.0
C9—C8—H8	119.2	C30—C31—C32	119.8 (2)
C7—C8—H8	119.2	C30—C31—H31	120.1
C8—C9—C10	120.0 (3)	C32—C31—H31	120.1
C8—C9—H9	120.0	C27—C32—C31	121.2 (2)
C10—C9—H9	120.0	C27—C32—H32	119.4
C11—C10—C9	119.5 (2)	C31—C32—H32	119.4
C11—C10—H10	120.3	Ni1—O33—H33A	105.4 (19)
C9—C10—H10	120.3	Ni1—O33—H33B	115.5 (17)
C10—C11—C12	120.1 (2)	H33A—O33—H33B	114 (3)
C10—C11—H11	119.9		

### Hydrogen-bond geometry (Å, º)

**Table:**

*D*—H···*A*	*D*—H	H···*A*	*D*···*A*	*D*—H···*A*
O13—H13*A*···O1^i^	0.84 (2)	1.95 (2)	2.747 (2)	159 (2)
O13—H13*B*···O23^ii^	0.86 (2)	1.88 (2)	2.658 (2)	150 (3)
O33—H33*A*···O3^iii^	0.83 (2)	1.88 (2)	2.691 (2)	163 (3)
O33—H33*B*···O21^iv^	0.83 (2)	1.97 (2)	2.748 (2)	156 (2)
N5—H5*B*···O1^i^	0.92	2.49	3.359 (2)	157
N5—H5*A*···O3^iii^	0.92	2.39	3.193 (3)	147
N25—H25*A*···O13^iv^	0.92	2.57	3.148 (2)	122
N25—H25*A*···O21^iv^	0.92	2.47	3.310 (2)	153
N25—H25*B*···O23^ii^	0.92	2.36	3.181 (3)	149

Symmetry codes: (i) *x*−1, *y*, *z*; (ii) *x*, *y*, *z*−1; (iii) *x*, *y*, *z*+1; (iv) *x*+1, *y*, *z*.
